# Effects of P-Coumarate 3-Hydroxylase Downregulation on the Compositional and Structural Characteristics of Lignin and Hemicelluloses in Poplar Wood (*Populus alba × Populus glandulosa*)

**DOI:** 10.3389/fbioe.2021.790539

**Published:** 2021-11-15

**Authors:** Xiao-Peng Peng, Jing Bian, Shuang-Quan Yao, Cheng-Ye Ma, Jia-Long Wen

**Affiliations:** ^1^ State Key Laboratory of Tree Genetics and Breeding, Key Laboratory of Tree Breeding and Cultivation of the National Forestry and Grassland Administration, Research Institute of Forestry, Chinese Academy of Forestry, Beijing, China; ^2^ Beijing Key Laboratory of Lignocellulosic Chemistry, Beijing Forestry University, Beijing, China; ^3^ Guangxi Key Laboratory of Clean Pulp and Papermaking and Pollution Control, College of Light Industry and Food Engineering, Guangxi University, Nanning, China

**Keywords:** C3H down-regulation, hemicelluloses, lignin, NMR, structural characteristics

## Abstract

Elucidating the chemical and structural characteristics of hemicelluloses and lignin in the *p*-coumarate 3-hydroxylase (C3H) down-regulated poplar wood will be beneficial to the upstream gene validation and downstream biomass conversion of this kind of transgenic poplar. Herein, the representative hemicelluloses and lignin with unaltered structures were prepared from control (CK) and C3H down-regulated 84K poplars. Modern analytical techniques, such as ^13^C NMR, 2D-HSQC NMR, and gel chromatography (GPC), were performed to better delineate the structural changes of hemicelluloses and lignin caused by transgenesis. Results showed that both the hemicelluloses (H_-CK_ and H_-C3H_) extracted from control and C3H down-regulated poplar wood have a chain backbone of (1→4)-β-D-Xylan with 4-*O*-Me-α-D-GlcpA as side chain, and the branch degree of the H_-C3H_ is higher than that of H_-CK_. With regarding to the lignin macromolecules, NMR results demonstrated that the syringyl/guaiacyl (S/G) ratio and dominant substructure β-*O*-4 linkages in C3H down-regulated poplar were lower than those of control poplar wood. By contrast, native lignin from C3H down-regulated poplar wood exhibited higher contents of *p*-hydroxybenzoate (PB) and *p*-hydroxyphenyl (H) units. In short, C3H down-regulation resulted in the chemical and structural changes of the hemicelluloses and lignin in these poplar wood. The identified structures will facilitate the downstream utilization and applications of lignocellulosic materials in the biorefinery strategy. Furthermore, this study could provide some illuminating results for genetic breeding on the improvement of wood properties and efficient utilization of poplar wood.

## Introduction

With the consumption of petrochemical resources and environmental concerns related to global warming and pollution, the search and development of renewable alternatives to petroleum-based resources have gained worldwide attraction (Himmel et al., 2007; Ragauskas et al., 2014). Lignocellulosic biomass represents a readily available renewable feedstock with the potential to be converted into a variety of fuels and chemicals (Ragauskas et al., 2006). Lignocellulosic biomass consists of three main components: lignin, hemicelluloses, and cellulose (Wang et al., 2019). Cellulose is a homopolymer which accounts for 30–50 wt% in lignocellulose, consisting of *β*-D-glucopyranose units linked by glycosidic bonds. Meanwhile, cellulose can be hydrolyzed enzymatically or chemically to obtain glucose, which can be further used to produce bioethanol and platform chemicals (Ma et al., 2021b). Hemicelluloses are amorphous polymers (15–30 wt% of lignocellulosic biomass) and consisted of C5 and C6 sugars. Due to the higher reactive activation than cellulose, hemicelluloses are easier to remove from lignocellulose to produce furfural and related chemicals (Peng et al., 2009). In addition, lignin is composed of aromatic monomers, which is an amorphous polymer accounting for 15–30 wt% in biomass (Wen et al., 2013b; Ragauskas et al., 2014).

Lignocelluloses are the largest renewable resource on Earth, which are considered to replace fossil-based products to produce chemicals, energy product, and fuels as the ideal raw materials (Sanderson, 2011; Isikgor and Becer, 2015; Wang et al., 2020). For a long time, lignocellulosic biomass has been considered a potential sustainable mixed sugar source, which can be used to ferment biomaterials and biofuels. (Ragauskas et al., 2014). However, “biomass recalcitrance” is created by tight binding of cellulose, hemicelluloses, and lignin, which is also the major obstacle for biorefinery (Himmel et al., 2007). The high cost of lignocellulose conversion is largely due to “biomass recalcitrance” (Zhao et al., 2012). Cellulose is difficult to be enzymatically hydrolyzed without pretreatment in woody biomass, which results from the existence of “biomass recalcitrance” (Ding et al., 2012; Sun et al., 2016). Ding et al. also pointed out that the ideal pretreatment should involve removing lignin as much as possible and reducing the modification of polysaccharides (Ding et al., 2012). “Biomass recalcitrance” must be reduced through pretreatment (Zhu and Pan, 2010; Pu et al., 2013). The second generation of biotechnological biofuel is liquid fuels (*e.g*., ethanol et al.) (Stephanopoulos, 2007; de Souza et al., 2014). The engineering feedstock crops will cost-competitively take the place of the fossil fuels to produce biofuels due to susceptible pretreatment and hydrolysis (Zhou et al., 2011). Lignin is the most abundant natural aromatic in plants because of its vital biological functions such as water retention and mechanical support. However, lignin can also inhibit saccharification by adsorbing hydrolytic enzymes (Alvira et al., 2010; Zheng et al., 2021). If lignin content and components can be reduced and altered by inhibiting the expression of critical genes in the lignin biosynthetic pathway, it will improve the efficiency of biorefinery and lower the cost (Sikarwar et al., 2016).

Lignin is a natural aromatic polymer composed of sinapyl alcohols, coniferyl, and hydroxycinnamyl (Vanholme et al., 2012a; Rinaldi et al., 2016; Vanholme et al., 2019; Zhao et al., 2020; Yang et al., 2021). The establishment of a suitable mass flux in the lignin biosynthesis pathway has become a new strategy for modifying lignin content (Boerjan et al., 2003). Realization of this strategy requires a comprehensive knowledge of lignin biosynthesis (Simmons et al., 2010). Researchers have made tremendous efforts to tailor the composition, structures, and reactivity of lignocellulose, especially lignin (Pilate et al., 2002). The lignin structure, composition, and content may vary among plant species and individuals, and even tissues of the same individual plant. Lignin biosynthesis is a complex process common to all vascular plants (Peng et al., 2014). Fortunately, the genes involved in this pathway have been studied and homologous genes for respective key genes are also known. There have been performed on genome-wide, transcript-, protein-, and metabolite-level studies, as well as the regulatory cascade of upstream transcription factors of these gene families, especially in Arabidopsis and poplar (Vanholme et al., 2012a; Vanholme et al., 2012b). The important enzymes in the phenylpropanoid biosynthetic pathway are hydroxycinnamoyl CoA: P-coumarate 3-hydroxylase (C3H), which could divert the pathway away from H lignin and toward S and G lignin (Franke et al., 2002a; Franke et al., 2002b; Wagner et al., 2007; Ralph et al., 2012). However, the compositional and structural characteristics of hemicelluloses and lignin from C3H-downregulated hardwood (poplar) have not been systematically characterized and researched. Herein, the effects of C3H downregulation on poplar hemicelluloses and lignin structures were investigated to determine how the genetic modification affects the hemicelluloses and lignin structures.

In this study, coumaroyl shikimate 3-hydroxylase (C3H) was cloned and constructed into RNAi vectors. Meanwhile, poplar was transformed by the leaf-disc method. A total of C3H-RNAi transgenic lines were obtained and vegetatively propagated by cutting for each line in the greenhouse. To illustrate the effects of C3H downregulation on the compositional and structural characteristics of lignin and hemicelluloses in poplar wood (*Populus alba × Populus glandulosa*), representative hemicelluloses and lignin samples were firstly isolated, and modern analytical techniques (high-performance anion exchange chromatography (HPAEC), ^13^C NMR, 2D-HSQC NMR, and gel chromatography (GPC) techniques) were applied to comprehensively delineate the chemical and structural changes of hemicelluloses and lignin caused by downregulation of the C3H gene. In short, this study is expected to provide some enlightenment for genetic breeding on the improvement of wood properties and efficient utilization of poplar wood in the current biorefinery engineering of woody biomass.

## Materials and methods

### Materials

Control 84K (CK) and downregulated C3H transgenic poplar 84K (*Populus alba × Populus glandulosa*, 4 years) were cultivated at the Chinese Academy of Forestry Sciences, and the detail regarding the procurement of wood was described in the ESI section. Especially, the gene-specific fragments were constructed into the RNAi plant expression vector by the double-digestion technique to obtain the RNAi expression vector of the C3H gene (Supplementary Figure S1). The *Populus alba × Populus glandulosa* clone 84K was used as transgenic poplar receptor material. Then, the resistant plants were obtained by the Agrobacterium tumefaciens-mediated transformation of leaf disc. That includes Agrobacterium culture, infection, coculture, selective culture, screening medium, and rooting culture (Supplementary Figure S2). The transplanted greenhouse was identified by PCR after the NPT-II gene and the target gene fragment, and the greenhouse was cut and propagated at low temperature and then the transgenic plants were obtained. The poplars were debarked and smashed into small pieces, then sieved to obtain 40–60 mesh particles. The composition of CK and C3H transgenics was determined by the National Renewable Energy Laboratory (NREL) standard analytical procedure (Sluiter et al., 2008). All the chemicals used in the experiment were of analytical grade.

### Determination of Klason lignin content

The determination of the Klason lignin content of CK and C3H poplar particles was based on the NREL standard analytical procedure (Sluiter et al., 2008). In detail, 0.3 g poplar sample was added to 72% H_2_SO_4_ to hydrolyze at 30°C for 1 h. Then, the solution was added with 84 ml deionized water for further hydrolysis at 121°C for 1 h. After the hydrolysis, the solution was filtrated using a G3 sand core funnel. The mass change before and after filtration was the weight of the Klason lignin.

### Isolation of representative hemicelluloses

Firstly, the control and C3H-downregulated poplar were delignified with acidic sodium chlorite solution (adjusted by acetic acid, pH 3.8–4.0) at 75°C for 2 h according to an earlier described procedure (Bian et al., 2012). As shown in Supplementary Figure S3, the delignified material (holocellulose) was extracted with 10% potassium hydroxide for 10 h (1:20 g ml^−1^) at room temperature. The liquid fractions were collected and adjusted to pH 5.5–6.0 with acetic acid. Then, the neutral solution was concentrated and precipitated in ethanol (three equivalent volumes). After filtration, the precipitates were redissolved in distilled water and dialyzed against water. After the freeze-dried process, the KOH-extracted hemicelluloses were obtained (named H_-CK_ and H_-C3H_, respectively).

### Preparation of representative native lignin

To delineate the structural characteristics of the native lignin in the raw material, double enzymatic lignins (DELs) from CK and C3H were prepared. The detailed preparation process was according to our previous publications (Chen et al., 2017a; Ma et al., 2020). As shown in Supplementary Figure S4, the ball-milled powder (5 g) was mixed with the desired amount of sodium acetate buffer (pH 4.8) with a solid-to-liquid ratio of 1:20 (g/ml) and cellulase (35 FPU/g substrate). Then, the mixture was incubated at 50°C in a rotary shaker with a rotational velocity of 150 rpm for 48 h. Next, the mixture was centrifuged and the residue was washed thoroughly with sodium acetate buffer (pH 4.8) to remove the hydrolyzed carbohydrates and then freeze-dried. Finally, the dried residual solid was repeatedly subjected to ball-milling for 2 h and enzymatic hydrolysis again, as in the abovementioned processes. After washing with acidic water (pH 2.0) and freeze-drying, DEL samples were obtained (named DEL_-CK_ and DEL_-C3H_). To increase the solubility of lignin in tetrahydrofuran (THF) for the determination of molecular weights by GPC technique, the acetylation of lignin was performed as previously described (Wang et al., 2017). All experiments in this study were conducted in duplicate, and the results reported were the average values.

### Methods

Sugar analysis (neutral sugars and uronic acids) was conducted by high-performance anion-exchange chromatography (HPAEC) in a Dionex ICS-3000 system (Dionex Corporation, Sunnyvale, CA, USA) equipped with a CarboPac PA1 (4 × 250 mm) column. The weight-average (M_w_) and number-average (M_n_) molecular weights of the samples were determined by gel permeation chromatography (GPC) (Agilent 1200, Agilent Technologies, Santa Clara, CA, USA) with an ultraviolet (UV) detector at 240 nm. The column used was a PL-gel 10 mm mixed-B 7.5 mm i.d. column, which was calibrated with PL polystyrene standards according to a previous report (Chen et al., 2017b). The NMR spectra of the samples were recorded at 25°C in DMSO-*d*_6_ (or D_2_O) on a Bruker AVIII 400 MHz spectrometer according to published procedures (Wen et al., 2013b; Wen et al., 2014). In detail, about 25 mg of lignin and hemicelluloses was dissolved in 0.5 ml of DMSO-*d*_6_ and D_2_O, respectively. For quantitative 2D-HSQC spectra, the Bruker standard pulse program hsqcetgpsi was used for HSQC experiments. The spectral widths were 5,000 Hz and 20,000 Hz for the ^1^H- and ^13^C-dimensions, respectively. The number of collected complex points was 1,024 for ^1^H-dimension with a recycle delay of 1.5 s. The number of transients was 64, and 256-time increments were always recorded in the ^13^C-dimension. The ^1^ *J*_CH_ used was 145 Hz. Prior to Fourier transformation, the data matrixes were zero filled up to 1,024 points in the ^13^C-dimension. Data processing was performed using standard Bruker Topspin-NMR software.

## Results and discussion

### Transcriptional abundance, plant height, and composition analysis

The inhibitory intensity of the gene is determined by detecting the expression level of the target gene at the transgenic plant. The expression of the transgene gene seriously affects the analysis of the subsequent result. Therefore, it is very important to detect the expression level of the transgenic gene. There are many ways to identify plant transgene expression at the transcriptional level, and a method for determining its specific mRNA is usually used. As shown in Supplementary Figure S5, the transcription level of the C3H gene in transgenic poplar was decreased in comparison with wild-type poplar (0.27). The expression of transgenic poplar was significantly reduced, and the average reduction was about 80%, indicating that the expression of the C3H gene was inhibited in transgenic CK.

**FIGURE 1:**
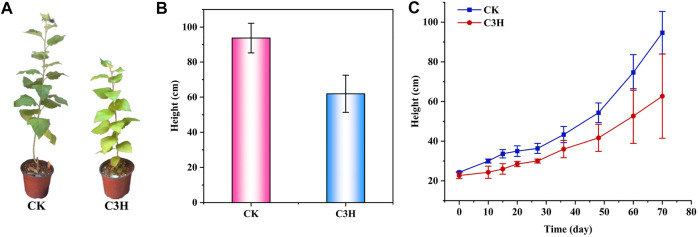
**(A)** Phenotypes of C3H transgenic plants and wild-type (CK) poplar plants. **(B)** and **(C)** Plant heights of C3H transgenic plants and wild-type (CK) plants.

According to Figure 1B, the plant height of wild-type plants (CK) was between 83 and 104 cm, the plant height of transgenic plants (C3H) was between 39 and 82 cm, and the average plant height was 61.93 cm. It was found that the height of transgenic poplars was lower than that of the CK, and the stems were browned to some extent (Figures 1A–C). Simultaneously, the results showed that the transgenic poplar woods had a significant decrease at different growth stages as compared to CK, and the height of C3H poplar decreased by about 34% under the same growing period. When the lignin synthetase was inhibited, it was bound to affect the growth of plant height, because lignin played a certain role in mechanical support. Hu and coauthors found that 4CL was a key gene lying upstream of C3H to adjust the lignin content (Hu et al., 1999). The 4CL suppression resulted in as much as a 45% reduction in total cell wall lignin and reportedly no impairment in growth (Hu et al., 1999). On the contrary, reductions in C3H resulted in varying effects on growth properties (Coleman et al., 2008). In this study, it was found that C3H downregulation could lead to the impairment in growth, which resulted in the relatively short plant height of C3H down-regulated poplar as compared to that of control (CK).

As shown in Table 1, regarding the chemical composition of CK and C3H, it was found that the content of Klason lignin in CK poplar wood was 25.94%, while the content of lignin was slightly decreased to 20.52% in C3H, which was slightly inconsistent with the previous reports (Pu et al., 2009; Ralph et al., 2012; Ma et al., 2021a). The reason for that was that C3H downregulation will inhibit the synthesis of lignin (Peng et al., 2016). By contrast, the contents of hemicelluloses and cellulose in the C3H-downregulated poplar woods slightly increased as compared to those of wild poplar (CK). In short, compositional analysis indicated that downregulation of C3H resulted in the slight changes of chemical composition of poplar wood, such as the decrease in lignin content.

**TABLE 1:** Composition analysis of CK and C3H.

Sample	Lignin^a^	Cellulose	Hemicelluloses
CK	25.94 ± 0.21	42.94 ± 0.32	22.15 ± 0.38
C3H	20.52 ± 0.38	44.29 ± 0.44	23.74 ± 0.42

a Lignin, Klason lignin.

### Effects of C3H downregulation on the compositional and structural characteristics of hemicelluloses

#### Monosaccharide analysis of the hemicelluloses

To survey the structural differences of the hemicelluloses during the C3H downregulation process, the scheme of hemicellulose isolation is illustrated in Supplementary Figure S3. Hemicelluloses are consisted of pentose and hexose, such as arabinose, rhamnose, glucose, xylose, mannose, galactose, and a small amount of glucuronic acid and galacturonic acid (Peng et al., 2012; Peng et al., 2009). Table 2 shows the neutral sugar and uronic acid contents of the CK and C3H hemicellulose samples. As illustrated in Table 2, the main glycosyl units of the hemicellulose were xylose (85.43%–85.61%), containing glucose (1.12%–1.72%), mannose (2.90%–3.15%), uronic acid (3.21%–4.41%), and a little of rhamnose (2.90%–2.99%), arabinose (1.02%–1.31%), and galactose (2.04%–2.19%), respectively. The xylose content of H_-CK_ and H_-C3H_ was 85.43% and 85.61%, respectively. Additionally, these hemicelluloses also contain 3.21% and 4.41% uronic acid (UA), indicating that H_-CK_ and H_-C3H_ mainly belonged to glucuronic acid-type xylan, and the other glycosyl groups can serve as a side chain attached to the main chain. A recent publication regarding the hardwood hemicelluloses demonstrated that hemicelluloses from hardwood were mainly composed of xylan-type and small amounts of mannan-type hemicelluloses (Qaseem et al., 2021). However, in this study, the monosaccharide analysis of the hemicelluloses showed that KOH-extracted hemicelluloses were principally the xylan-type hemicelluloses. This discrepancy is probably related to the tree species and the extraction method of hemicelluloses. However, the detailed composition and structural features of the hemicelluloses still need to be confirmed *via* NMR characterization. Additionally, the ratio of uronic acid to xylose (UA/Xyl) can contribute to understanding the degree of linearity or branching of hemicelluloses (Wen et al., 2010; Peng et al., 2012). It can be seen from the uronic acid/xylose ratio that H_-C3H_ (UA/Xyl, 0.04) had a more linear chain than H_-CK_ (UA/Xyl, 0.05), implying that hemicelluloses from C3H-downregulated poplar wood had more linear structures. However, the differences in monosaccharide components of hemicelluloses from CK and C3H were not particularly pronounced. The structural characteristics of the hemicellulose from CK and C3H down-regulated poplar wood samples will be discussed in the following NMR analysis.

**TABLE 2:** Monosaccharide content of the KOH-extracted hemicelluloses.

Sample	Molar composition (%)							
	Rha^a^	Ara	Gal	Glu	Xyl	Man	UA	UA/Xyl
H_-CK_	2.99	1.31	2.19	1.72	85.43	3.15	3.21	0.04
H_-C3H_	2.90	1.02	2.04	1.12	85.61	2.90	4.41	0.05

a Abbreviation: Rha, rhamnose; Ara, arabinose; Gal, galactose; Glu, glucose; Xyl, xylose; UA, uronic acid.

#### Molecular weights and NMR analysis of the hemicelluloses

The weight-average molecular weight (M_w_), number-average molecular weight (M_n_), and polydispersity (M_w_/M_n_) of the hemicelluloses are shown in Table 3. The M_w_ of H_-CK_ and H_-C3H_ were 30,720, and 39,800 g/mol, indicating that the M_w_ of H_-CK_ was less than that of H_-C3H_. The polydispersity index (PDI) of H_-CK_ (2.10) was higher than that of H_-C3H_ (1.88), implying that H_-C3H_ has a narrow molecular weight distribution and H_-C3H_ exhibits a relatively homogeneous structure.

**TABLE 3:** Weight-average (M_w_) and number-average (M_n_) molecular weights and polydispersity (M_w_/M_n_) of the hemicelluloses and lignin fractions.

	H_-CK_	H_-C3H_	DEL_-CK_	DEL_-C3H_
M_w_	30,720	39,800	8,020	7,410
M_n_	14,600	21,150	4,220	4,080
M_w_/M_n_	2.10	1.88	1.90	1.81

**FIGURE 2:**
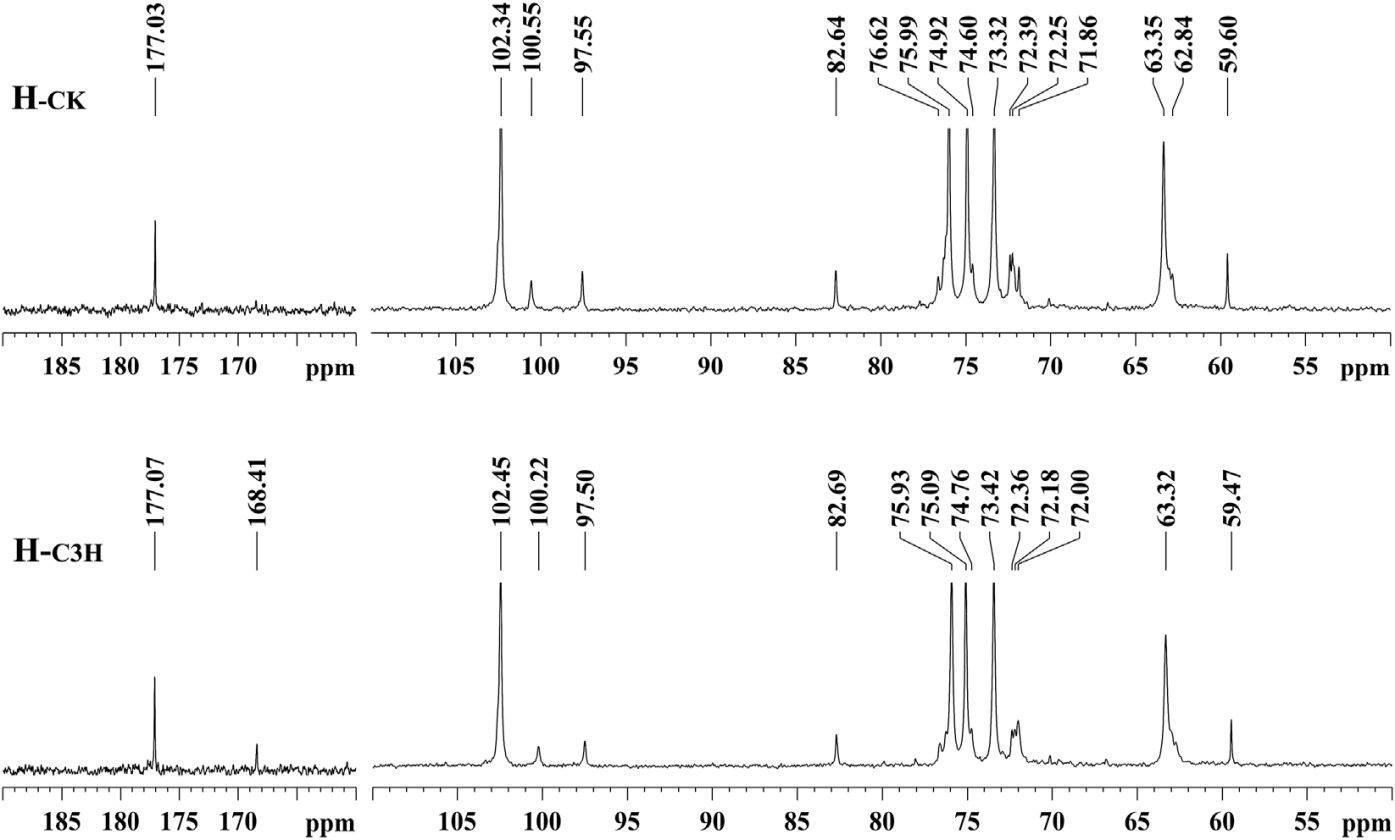
^13^C NMR spectra of the hemicelluloses.

For further understanding of the structural characteristics of hemicelluloses, H_-CK_ and H_-C3H_ were characterized by NMR techniques (Peng et al., 2010; Wen et al., 2010; Bian et al., 2012). The NMR techniques can obtain valuable information about the backbone of the hemicelluloses and their branching side chains. Figure 2 shows the ^13^C NMR spectra of the CK and C3H hemicelluloses (H_-CK_ and H_-C3H_). For ^13^C NMR spectra, all hemicelluloses had strong signals at 74.92, 63.35, 75.99, 73.32, and 102.34 ppm, which were characteristic of the C-3, C-5, C-4, C-2, and C-1 positions of the (1→4)-linked-*β*-D-xylopyranoside units. Additionally, the -OCH_3_, C-2, C-4, C-3, C-1, C-6, and C-5 of the 4-*O*-methyl-α-glucuronic acid units were located at 59.60, 71.86, 82.64, 72.39, 97.55, 177.03, and 72.25 ppm, respectively. The hemicelluloses from CK and C3H-downregulated poplar wood exhibited similar chemical shifts, suggesting that these hemicelluloses had the same structural characteristics. Especially, as compared with H_-CK_, the ^13^C NMR spectra of H_-C3H_ showed a weak signal at 168.41 ppm, which was the characteristic signal of the free *p*-hydroxybenzoic acid (PB). This phenomenon suggested that the C3H-downregulated poplar contained more *p*-hydroxybenzoic acid, which will also be demonstrated by 2D-HSQC NMR below. In fact, C3H poplar wood contained more PB units (especially for lignin); thus, a bit of *p*-hydroxybenzoic acid in the cell wall after KOH extraction was co-precipitated with the hemicelluloses.

**FIGURE 3:**
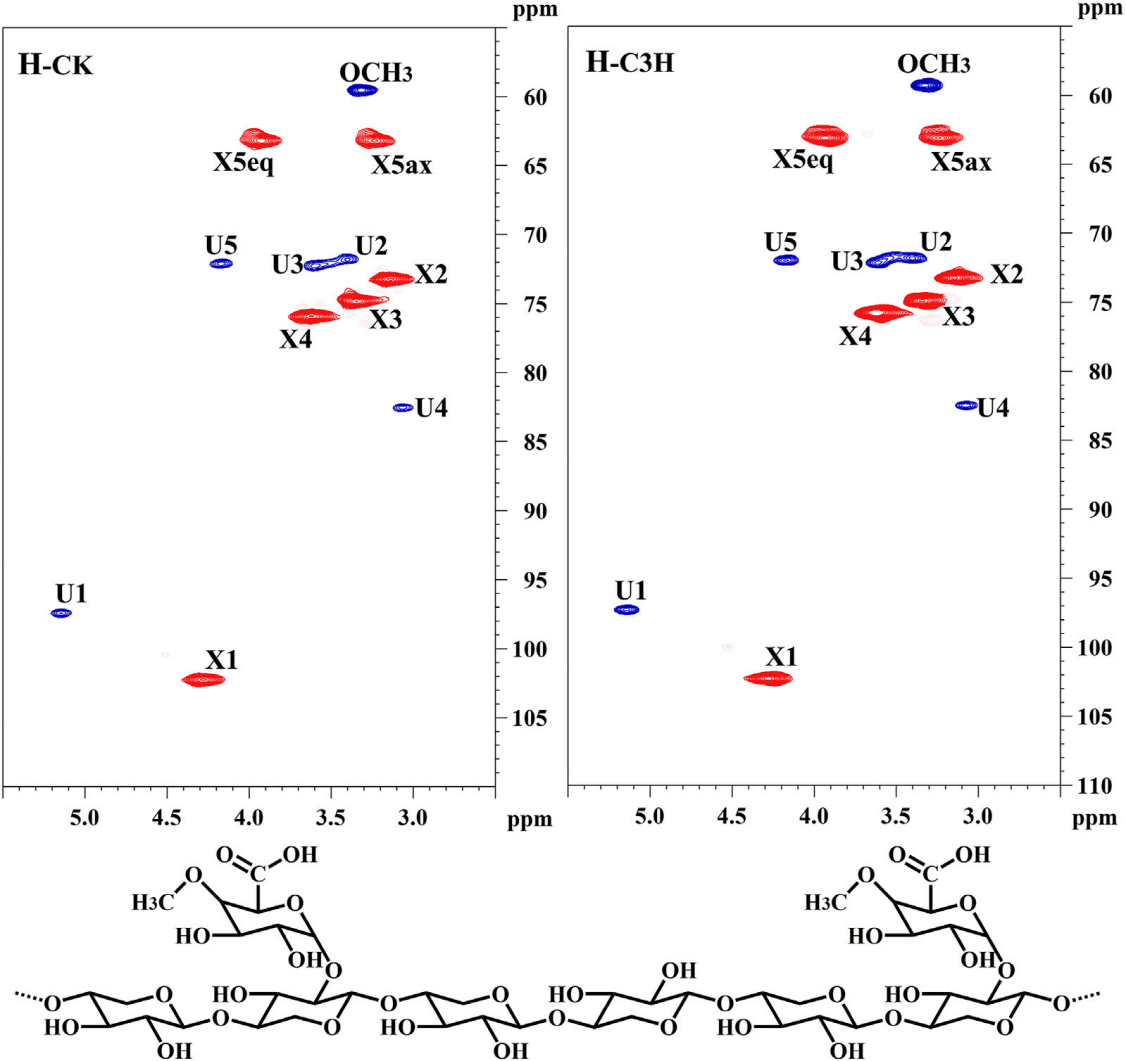
2D-HSQC spectra and conjectural structures of the hemicelluloses.

To further uncover the molecular structural characteristics of H_-CK_ and H_-C3H_, the 2D-HSQC NMR spectral analysis of these hemicelluloses was performed and the spectra were assigned according to the previous publication (Wen et al., 2010). As shown in Figure 3, the prominent signals corresponding to the (1→4)-*β*-D-Xylp backbone and 4-*O*-Me-*α*-D-GlcpA side chain in all the spectra were found. Especially, the main cross-peaks of C_1_-H_1_, C_4_-H_4_, C_3_-H_3_, C_2_-H_2_, and C_5_-H_5_ of the (1→4)-linked-*β*-D-Xylp units were distributed at _δ_C/_δ_H 102.2/4.28, 76.0/3.63, 75.0/3.34, 73.1/3.13, 63.2/3.93, and 3.27. Additionally, a distinguishable cross-peak at 60.1/3.32 was assigned to the methoxy group (-OCH_3_) in 4-*O*-methyl-D-glucuronic acid. For 4-*O*-methyl-D-glucuronic acid, the signals appear at _δ_C/_δ_H 97.31/5.14 (C_1_-H_1_) and _δ_C/_δ_H 71.6/3.41 (C_2_-H_2_), _δ_C/_δ_H 72.16/3.62 (C_3_-H_3_), _δ_C/_δ_H 82.48/3.07 (C_4_-H_4_), _δ_C/_δ_H 72.0/4.20 (C_5_-H_5_) (Yuan et al., 2010). According to the existing literature concerning the NMR linkages between the monosaccharides (Peng et al., 2010), it could be found that the KOH-extracted hemicelluloses from these poplar woods were mainly composed of a linear backbone of (*β*-1–4)-Xylp residues, and the xylose was substituted by 4-*O*-methyl-*α*-D-GlcpA at the C_2_ position. Based on the results of NMR and sugar analysis of the hemicelluloses, it was suggested that H_-CK_ and H_-C3H_ were mainly composed of the 4-*O*-Me-α-D-GlcpA side chains attached to a linear backbone of (1→4)-β-D-Xylp.

### Effects of C3H downregulation on the molecular weights and structural characteristics of native lignin

In fact, C3H downregulation mainly affects the biosynthesis of the lignin macromolecule. Herein, the effects of C3H downregulation on the molecular weights and structural characteristics of native lignin were investigated and discussed in detail. The M_w_ and M_n_ and polydispersity index (M_w_/M_n_) of DEL_-CK_ and DEL_-C3H_ are displayed in Table 3. The M_w_ of DEL_-CK_ and DEL_-C3H_ was 8,020 and 7,410 g/mol, respectively. The higher M_w_ of lignin was partly related to the relatively high β-*O*-4 content, as observed previously (Wen et al., 2013). DEL_-C3H_ had relatively lower molecular weight distributions (1.81) as compared to DEL_-CK_ (1.90), implying that the downregulation of C3H facilitates the formation of relatively homogeneous lignin fractions. This phenomenon was similar to that of hemicellulose, implying that downregulation of C3H led to the homogenization of biomacromolecules.

**FIGURE 4:**
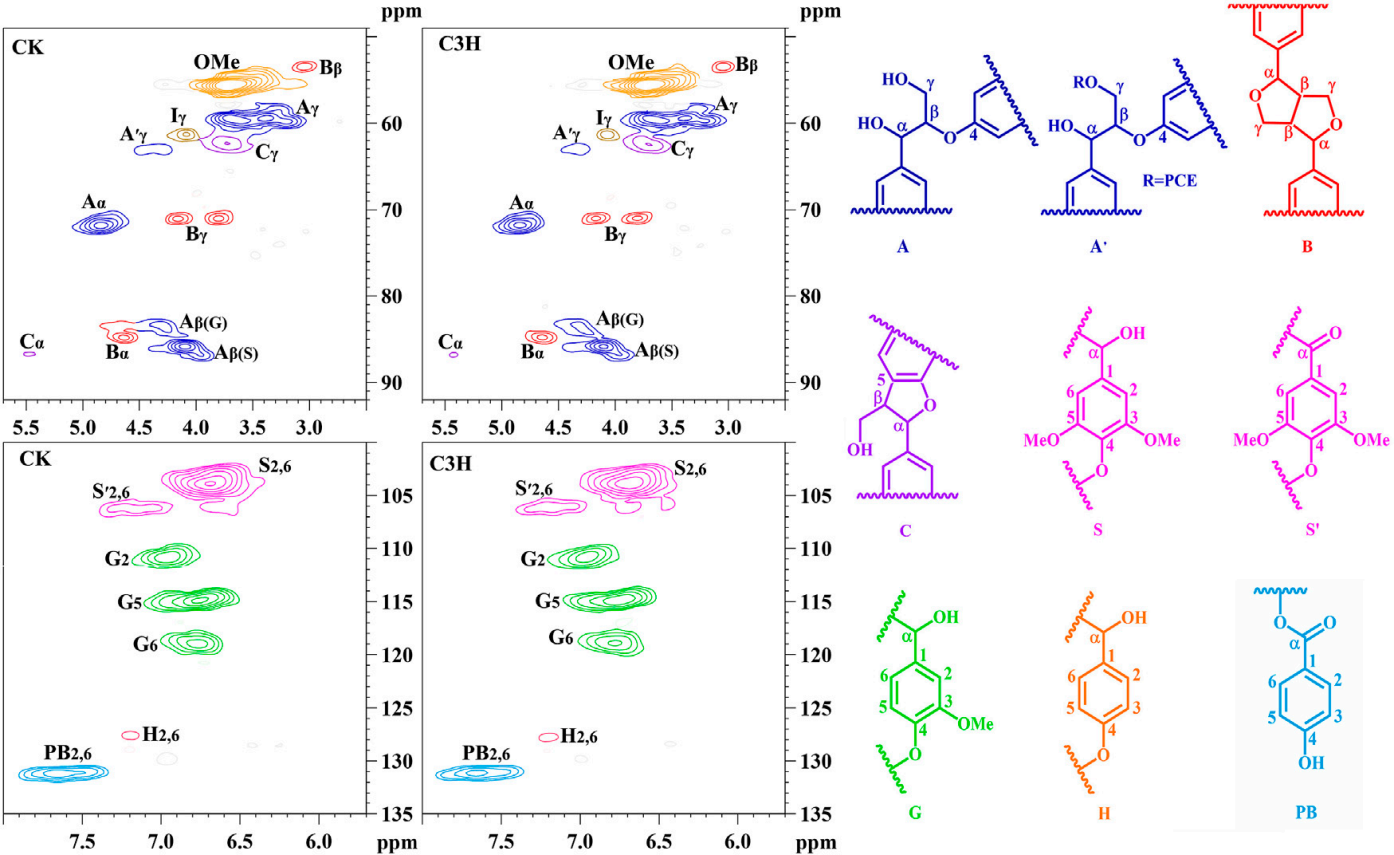
2D-HSQC spectra and identified main structures of the lignin fractions isolated from poplar wood.

To demonstrate the structural differences of native lignin between control 84K poplar (CK) and C3H-downregulated poplar (C3H) samples, the lignin samples were analyzed by the 2D HSQC NMR technique. These differences could provide some fundamental basis for obtaining ideal lignin sources for subsequent lignin valorization (Wen et al., 2013a). Figure 4 shows the chemical composition (aromatic region) and interunit linkages (side-chain region) in the 2D-HSQC spectra of DEL_-CK_ and DEL_-C3H_ according to the previous signal assignments (Wen et al., 2015). In the side-chain regions (_δ_C/_δ_H 49–92/2.5–5.7) of the 2D-HSQC spectra, the linkages of β-*O*-4 aryl ethers (A), resinols (B), and phenylcoumarans (C) could be obviously observed. It was found that DEL_-C3H_ and DEL_-CK_ exhibited similar but discriminative spectral patterns. Cross-signals of β-*O*-4 and -OCH_3_ (_δ_C/_δ_H 55.7/3.70) were the prominent signals. _δ_C/_δ_H 71.9/4.87 were cross-signals of C_α_-H_α_ correlations in the β-*O*-4 linkages, while the β-*O*-4 linkages (C_β_-H_β_) linked to G and S units can be distinguished at _δ_C/_δ_H 83.5/4.34 and 85.7/4.12. _δ_C/_δ_H 59.5/3.71–3.40 was assigned C_γ_-H_γ_ correlations in the β-*O*-4 substructures. Meanwhile, the content of β-*O*-4 linkages in DEL_-C3H_ was higher than that of DEL_-CK_, which was consistent with the results in a previous publication (Ma et al., 2021a). In addition, _δ_C/_δ_H 62.9/4.40 was assigned C_γ_-H_γ_ correlations in γ-acylated lignin units (A′). This indicated that those lignin samples were partially γ-carbon acylated in β-*O*-4 aryl ether linkages and *p*-hydroxybenzoates (PB). In a recent publication, whether *p*-hydroxybenzoates acylate solely S units in transgenics poplar has not been confirmed (Ralph et al., 2012). Resinols (β-β, substructures B) can be easily identified in the spectra in conspicuous amounts. _δ_C/_δ_H 84.8/4.67, 53.4/3.04, and 71.1/3.80–4.19 were assigned their C_α_-H_α_, C_β_-H_β_ and the double C_γ_-H_γ_ correlations, respectively. The weak signal of C_α_-H_α_ correlations of phenylcoumarans (β-5, _δ_C/_δ_H, 86.8/5.49) suggested that the low content of β-5 linkages (DEL_-CK_ 1.18/100Ar, DEL_-C3H_ 1.14/100Ar). This phenomenon could be attributed to the reduction of G units (relative to per 100Ar) as compared to that of CK based on a publication (Wang et al., 2018), in which it was reported that phenylcoumaran (β-5) was derived from the coupling of a monolignol with G units.

In the aromatic regions (_δ_C/_δ_H 100–135/5.7–8.0) of the 2D-HSQC NMR spectra (Figure 4), the chemical composition in the lignin samples (DEL_-CK_ and DEL_-C3H_) can be clearly observed, such as syringyl (S) and guaiacyl (G) lignin units and some other lignin substructures. The C_2,6_-H_2,6_ correlation at _δ_C/_δ_H 103.8/6.68 represented the prominent signal for S-type lignin units, whereas the signal at _δ_C/_δ_H 106.2/7.18 was observed for the C_α_-oxidized S-units (S′). Additionally, the different correlations of C_2_-H_2_ (_δ_C/_δ_H 110.9/6.97), C_5_-H_5_ (_δ_C/_δ_H 114.8/6.77), and C_6_-H_6_ (_δ_C/_δ_H 118.8/6.78) belonged to G-type lignin units. Specially, H_2,6_ signals were detected at _δ_C/_δ_H 127.7/7.15, which increased from 0.6 to 1.1/100Ar, suggesting a striking elevation of *p*-hydroxyphenyl (H) units in transgenic poplar. The relative abundances of different linkages in lignin were quantified according to the previous literatures (Ma et al., 2021a; Wen et al., 2013a). The changes of the S/G ratio can intuitively reflect the compositional change of lignin samples. As is shown in Table 4, the S/G ratio in DEL_-CK_ was 2.82, while the S/G ratio for DEL_-C3H_ was 2.48. Interestingly, the relative content of H-type lignin in DEL_-C3H_ (1.11/100Ar) was higher than that in DEL_-CK_ (0.6/100Ar). This fact suggested that H-type lignin units have been elevated in C3H-downregulated poplar. Similarly, this phenomenon had been reported in a previous study, in which the increased amount of *p*-hydroxyphenyl unit was observed as well as a concomitant decrease of guaiacyl and syringyl units (Pu et al., 2009). In this study, the spectra shown in Figure 4 clearly showed the enhancement of PB content in DEL_-C3H_. Precisely, the integral value of PB increased from 14.17 to 16.37/100Ar (as shown in Table 4), which was in agreement with a previous study (Ralph et al., 2012). In short, the C3H downregulated could increase the content of H units and *p*-hydroxybenzoate (PB) units in lignin. These results were also in line with the ^13^C-NMR section of H-_C3H_, in which more PB units were detected. In short, 2D-HSQC spectra of native lignin and hemicellulose samples demonstrated that C3H downregulation indeed altered the chemical and structural features of these natural macromolecules to different extents. Due to these differences in composition and structure, the processing performance of transgenic poplar wood will be affected and the corresponding investigations are being explored.

**TABLE 4:** Quantification of lignin fractions by quantitative 2D-HSQC NMR spectroscopy.

Sample	DEL_-CK_	DEL_-C3H_
β-*O*-4^a^	57.79	56.78
β-β	13.02	10.76
β-5	1.18	1.14
PB	14.17	16.37
S/G^b^	2.82	2.48
S/G/H	73.4/26/0.6	70.5/28.4/1.1

a Result expressed per 100 Ar based on quantitative 2D-HSQC spectra.

b S/G ratio obtained by this equation: S/G ratio = 0.5I (S_2,6_)/I (G_2_).

### Implications

The elevated lignin in the *p*-hydroxyphenyl (H) unit is produced by the downregulation of C3H in poplar wood, and the lignin content is also significantly reduced. A previous study investigated the effects of C3H downregulation on the lignin in alfalfa (Ralph et al., 2006). Ralph and coauthors found that the lignins rich in *p*-hydroxyphenyl units were produced by C3H downregulation, but the S/G ratio changed only slightly in alfalfa. Conversely, the S/G ratio of lignin was increased in the C3H downregulation poplar. Most of the relative H unit elevation was at the expense of G units rather than S units in poplar (Ralph et al., 2012). In general, genetic modification during lignin biosynthesis led to dwarfing or developmental abnormalities of the transgenic plants (Bonawitz and Chapple, 2013). However, with the growth and development of the plant, the transgenic poplar probably restores growth if there is an active cell wall feedback signaling responsible for dwarfing existing in lignin-deficient mutants (Bonawitz and Chapple, 2013). Simultaneously, as a pendant group, the PB content in lignin was increased in C3H poplar as compared to CK wood. However, the related transferase in poplar has not been identified. The identified transferase will help to understand how the transgenes affect the pendant group, such as *p*-hydroxybenzoates in hardwood and *p*-coumarate in gramineous plants.

## Conclusion

In this study, the representative alkaline hemicelluloses (KOH hemicelluloses) and lignin (double enzymatic lignin, DEL) were respectively extracted from control (CK) and C3H-downregulated 84K (C3H) poplars, which can better characterize the structural variations of hemicelluloses and lignin macromolecules in control and C3H-downregulated poplars. Results showed that H_-CK_ and H_-C3H_ were mainly composed of a linear backbone of (1→4)-*β*-D-Xylp with 4-*O*-Me-α-D-GlcpA attached as side chain, and the branching degree of H_-CK_ was more than that of H_-C3H_. Meanwhile, the downregulation of C3H could decrease the lignin content. Results showed that native lignin of CK and C3H exhibited similar structural features; nevertheless, transgenic poplars had relatively lower contents of β-*O*-4 linkages and S/G ratios as well as a relatively higher content of H-type lignin units. Furthermore, the content of PB content in poplar wood was increased in the lignin from C3H-downregulated poplar. In short, understanding the structural characteristics of native hemicelluloses and lignin from control and transgenic poplar is conducive to selecting optimal hemicelluloses and lignin characteristics required for downstream applications and utilization of lignocellulosic materials in the biorefinery strategy.

## Data Availability

The original contributions presented in the study are included in the article/Supplementary Material; further inquiries can be directed to the corresponding authors.

## References

[B1] Alvira P. Tomás-Pejó E. Ballesteros M. Negro M. J. (2010). Pretreatment Technologies for an Efficient Bioethanol Production Process Based on Enzymatic Hydrolysis: A Review. Bioresour. Technol. 101 (13), 4851–4861. 10.1016/j.biortech.2009.11.093 20042329

[B2] Bian J. Peng F. Peng P. Xu F. Sun R.-C. (2012). Chemical Composition and Structural Feature of Populus Gansuensis Hemicellulosic Polymers. J. Appl. Polym. Sci. 124 (4), 3154–3164. 10.1002/app.34835

[B3] Boerjan W. Ralph J. Baucher M. (2003). Lignin Biosynthesis. Annu. Rev. Plant Biol. 54 (1), 519–546. 10.1146/annurev.arplant.54.031902.134938 14503002

[B4] Bonawitz N. D. Chapple C. (2013). Can Genetic Engineering of Lignin Deposition Be Accomplished without an Unacceptable Yield Penalty? Curr. Opin. Biotechnol. 24 (2), 336–343. 10.1016/j.copbio.2012.11.004 23228388

[B5] Chen T.-Y. Wang B. Wu Y.-Y. Wen J.-L. Liu C.-F. Yuan T.-Q. (2017a). Structural Variations of Lignin Macromolecule from Different Growth Years of Triploid of Populus Tomentosa Carr. Int. J. Biol. Macromolecules 101, 747–757. 10.1016/j.ijbiomac.2017.03.146 28363656

[B6] Chen T.-Y. Wen J.-L. Wang B. Wang H.-M. Liu C.-F. Sun R.-C. (2017b). Assessment of Integrated Process Based on Autohydrolysis and Robust Delignification Process for Enzymatic Saccharification of Bamboo. Bioresour. Technol. 244, 717–725. 10.1016/j.biortech.2017.08.032 28822283

[B7] Coleman H. D. Samuels A. L. Guy R. D. Mansfield S. D. (2008). Perturbed Lignification Impacts Tree Growth in Hybrid Poplar-A Function of Sink Strength, Vascular Integrity, and Photosynthetic Assimilation. Plant Physiol. 148 (3), 1229–1237. 10.1104/pp.108.125500 18805953 PMC2577275

[B8] de Souza R. O. M. A. Miranda L. S. M. Luque R. (2014). Bio(chemo)technological Strategies for Biomass Conversion into Bioethanol and Key Carboxylic Acids. Green. Chem. 16 (5), 2386–2405. 10.1039/c3gc41885e

[B9] Ding S.-Y. Liu Y.-S. Zeng Y. Himmel M. E. Baker J. O. Bayer E. A. (2012). How Does Plant Cell wall Nanoscale Architecture Correlate with Enzymatic Digestibility? Science 338 (6110), 1055–1060. 10.1126/science.1227491 23180856

[B10] Franke R. Hemm M. R. Denault J. W. Ruegger M. O. Humphreys J. M. Chapple C. (2002a). Changes in Secondary Metabolism and Deposition of an Unusual Lignin in the Ref8 Mutant of Arabidopsis. Plant J. 30 (1), 47–59. 10.1046/j.1365-313x.2002.01267.x 11967092

[B11] Franke R. Humphreys J. M. Hemm M. R. Denault J. W. Ruegger M. O. Cusumano J. C. (2002b). The Arabidopsis REF8 Gene Encodes the 3-Hydroxylase of Phenylpropanoid Metabolism. Plant J. 30 (1), 33–45. 10.1046/j.1365-313x.2002.01266.x 11967091

[B12] Himmel M. E. Ding S.-Y. Johnson D. K. Adney W. S. Nimlos M. R. Brady J. W. (2007). Biomass Recalcitrance: Engineering Plants and Enzymes for Biofuels Production. Science 315 (5813), 804–807. 10.1126/science.1137016 17289988

[B13] Hu W.-J. Harding S. A. Lung J. Popko J. L. Ralph J. Stokke D. D. (1999). Repression of Lignin Biosynthesis Promotes Cellulose Accumulation and Growth in Transgenic Trees. Nat. Biotechnol. 17 (8), 808–812. 10.1038/11758 10429249

[B14] Isikgor F. H. Becer C. R. (2015). Lignocellulosic Biomass: A Sustainable Platform for the Production of Bio-Based Chemicals and Polymers. Polym. Chem. 6 (25), 4497–4559. 10.1039/c5py00263j

[B15] Ma C.-Y. Gao X. Peng X.-P. Gao Y.-F. Liu J. Wen J.-L. (2021a). Microwave-Assisted Deep Eutectic Solvents (DES) Pretreatment of Control and Transgenic Poplars for Boosting the Lignin Valorization and Cellulose Bioconversion. Ind. Crops Prod. 164, 113415. 10.1016/j.indcrop.2021.113415

[B16] Ma C.-Y. Wang H.-M. Wen J.-L. Shi Q. Wang S.-F. Yuan T.-Q. (2020). Structural Elucidation of Lignin Macromolecule from Abaca during Alkaline Hydrogen Peroxide Delignification. Int. J. Biol. Macromolecules 144, 596–602. 10.1016/j.ijbiomac.2019.12.080 31837367

[B17] Ma C.-Y. Xu L.-H. Zhang C. Guo K.-N. Yuan T.-Q. Wen J.-L. (2021b). A Synergistic Hydrothermal-Deep Eutectic Solvent (DES) Pretreatment for Rapid Fractionation and Targeted Valorization of Hemicelluloses and Cellulose from Poplar Wood. Bioresour. Technol. 341, 125828. 10.1016/j.biortech.2021.125828 34461401

[B18] Peng F. Peng P. Xu F. Sun R.-C. (2012). Fractional Purification and Bioconversion of Hemicelluloses. Biotechnol. Adv. 30 (4), 879–903. 10.1016/j.biotechadv.2012.01.018 22306329

[B19] Peng F. Ren J.-L. Xu F. Bian J. Peng P. Sun R.-C. (2009). Comparative Study of Hemicelluloses Obtained by Graded Ethanol Precipitation from Sugarcane Bagasse. J. Agric. Food Chem. 57 (14), 6305–6317. 10.1021/jf900986b 19537731

[B20] Peng F. Ren J.-L. Xu F. Bian J. Peng P. Sun R.-C. (2010). Fractionation of Alkali-Solubilized Hemicelluloses from Delignified Populus Gansuensis: Structure and Properties. J. Agric. Food Chem. 58 (9), 5743–5750. 10.1021/jf1003368 20302372

[B21] Peng X.-P. Sun S.-L. Wen J.-L. Yin W.-L. Sun R.-C. (2014). Structural Characterization of Lignins from Hydroxycinnamoyl Transferase (HCT) Down-Regulated Transgenic Poplars. Fuel 134, 485–492. 10.1016/j.fuel.2014.05.069

[B22] Peng X.-P. Wang B. Wen J.-L. Yang S.-Z. Lu M.-Z. Sun R.-C. (2016). Effects of Genetic Manipulation (HCT and C3H Down-Regulation) on Molecular Characteristics of Lignin and its Bioconversion to Fermentable Sugars. Cellulose Chem. Technol. 50, 649–658.

[B23] Pilate G. Guiney E. Holt K. Petit-Conil M. Lapierre C. Leplé J.-C. (2002). Field and Pulping Performances of Transgenic Trees with Altered Lignification. Nat. Biotechnol. 20 (6), 607–612. 10.1038/nbt0602-607 12042866

[B24] Pu Y. Hu F. Huang F. Davison B. H. Ragauskas A. J. (2013). Assessing the Molecular Structure Basis for Biomass Recalcitrance during Dilute Acid and Hydrothermal Pretreatments. Biotechnol. Biofuels 6 (1), 15–13. 10.1186/1754-6834-6-15 23356640 PMC3575271

[B25] Pu Y. Chen F. Ziebell A. Davison B. H. Ragauskas A. J. (2009). NMR Characterization of C3H and HCT Down-Regulated Alfalfa Lignin. Bioenerg. Res. 2 (4), 198–208. 10.1007/s12155-009-9056-8

[B26] Qaseem M. F. Shaheen H. Wu A.-M. (2021). Cell Wall Hemicellulose for Sustainable Industrial Utilization. Renew. Sustain. Energ. Rev. 144, 110996. 10.1016/j.rser.2021.110996

[B27] Ragauskas A. J. Beckham G. T. Biddy M. J. Chandra R. Chen F. Davis M. F. (2014). Lignin Valorization: Improving Lignin Processing in the Biorefinery. Science 344 (6185), 1246843. 10.1126/science.1246843 24833396

[B28] Ragauskas A. J. Williams C. K. Davison B. H. Britovsek G. Cairney J. Eckert C. A. (2006). The Path Forward for Biofuels and Biomaterials. Science 311 (5760), 484–489. 10.1126/science.1114736 16439654

[B29] Ralph J. Akiyama T. Coleman H. D. Mansfield S. D. (2012). Effects on Lignin Structure of Coumarate 3-Hydroxylase Downregulation in Poplar. Bioenerg. Res. 5 (4), 1009–1019. 10.1007/s12155-012-9218-y PMC4560085 26366246

[B30] Ralph J. Akiyama T. Kim H. Lu F. Schatz P. F. Marita J. M. (2006). Effects of Coumarate 3-Hydroxylase Down-Regulation on Lignin Structure. J. Biol. Chem. 281 (13), 8843–8853. 10.1074/jbc.m511598200 16421107

[B31] Rinaldi R. Jastrzebski R. Clough M. T. Ralph J. Kennema M. Bruijnincx P. C. A. (2016). Paving the Way for Lignin Valorisation: Recent Advances in Bioengineering, Biorefining and Catalysis. Angew. Chem. Int. Ed. 55 (29), 8164–8215. 10.1002/anie.201510351 PMC6680216 27311348

[B32] Sanderson K. (2011). Lignocellulose: A Chewy Problem. Nature 474 (7352), S12–S14. 10.1038/474s012a 21697834

[B33] Sikarwar V. S. Zhao M. Clough P. Yao J. Zhong X. Memon M. Z. (2016). An Overview of Advances in Biomass Gasification. Energy Environ. Sci. 9 (10), 2939–2977. 10.1039/c6ee00935b

[B34] Simmons B. A. Loqué D. Ralph J. (2010). Advances in Modifying Lignin for Enhanced Biofuel Production. Curr. Opin. Plant Biol. 13 (3), 312–319. 10.1016/j.pbi.2010.03.001 20359939

[B35] Sluiter A. Hames B. Ruiz R. Scarlata C. Sluiter J. Templeton D. (2008). Determination of Structural Carbohydrates and Lignin in Biomass. Lab. Anal. procedure 1617 (1), 1–16.

[B36] Stephanopoulos G. (2007). Challenges in Engineering Microbes for Biofuels Production. Science 315 (5813), 801–804. 10.1126/science.1139612 17289987

[B37] Sun S. Sun S. Cao X. Sun R. (2016). The Role of Pretreatment in Improving the Enzymatic Hydrolysis of Lignocellulosic Materials. Bioresour. Technol. 199, 49–58. 10.1016/j.biortech.2015.08.061 26321216

[B38] Vanholme R. De Meester B. Ralph J. Boerjan W. (2019). Lignin Biosynthesis and its Integration into Metabolism. Curr. Opin. Biotechnol. 56, 230–239. 10.1016/j.copbio.2019.02.018 30913460

[B39] Vanholme R. Morreel K. Darrah C. Oyarce P. Grabber J. H. Ralph J. (2012a). Metabolic Engineering of Novel Lignin in Biomass Crops. New Phytol. 196 (4), 978–1000. 10.1111/j.1469-8137.2012.04337.x 23035778

[B40] Vanholme R. Storme V. Vanholme B. Sundin L. Christensen J. H. Goeminne G. (2012b). A Systems Biology View of Responses to Lignin Biosynthesis Perturbations in Arabidopsis. The Plant Cell 24 (9), 3506–3529. 10.1105/tpc.112.102574 23012438 PMC3480285

[B41] Wagner M. S. Wajner S. M. Dora J. M. Maia A. L. (2007). Regulation of Dio2 Gene Expression by Thyroid Hormones in Normal and Type 1 Deiodinase-Deficient C3H Mice. J. Endocrinol. 193 (3), 435–444. 10.1677/joe-07-0099 17535881

[B42] Wang H.-M. Wang B. Wen J.-L. Wang S.-F. Shi Q. Sun R.-C. (2018). Green and Efficient Conversion Strategy of Eucalyptus Based on Mechanochemical Pretreatment. Energ. Convers. Manage. 175, 112–120. 10.1016/j.enconman.2018.09.002

[B43] Wang H.-M. Wang B. Wen J.-L. Yuan T.-Q. Sun R.-C. (2017). Structural Characteristics of Lignin Macromolecules from Different Eucalyptus Species. ACS Sustain. Chem. Eng. 5 (12), 11618–11627. 10.1021/acssuschemeng.7b02970

[B44] Wang H.-M. Wang B. Yuan T.-Q. Zheng L. Shi Q. Wang S.-F. (2020). Tunable, UV-Shielding and Biodegradable Composites Based on Well-Characterized Lignins and Poly(Butylene Adipate-Co-Terephthalate). Green. Chem. 22 (24), 8623–8632. 10.1039/d0gc03284k

[B45] Wang H. Wang B. Sun D. Shi Q. Zheng L. Wang S. (2019). Unraveling the Fate of Lignin from Eucalyptus and Poplar during Integrated Delignification and Bleaching. ChemSusChem 12 (5), 1059–1068. 10.1002/cssc.201802592 30648348

[B46] Wen J.-L. Sun S.-L. Xue B.-L. Sun R.-C. (2013a). Quantitative Structural Characterization of the Lignins from the Stem and Pith of Bamboo (Phyllostachys Pubescens). Holzforschung 67 (6), 613–627. 10.1515/hf-2012-0162

[B47] Wen J.-L. Sun S.-L. Xue B.-L. Sun R.-C. (2013b). Quantitative Structures and Thermal Properties of Birch Lignins after Ionic Liquid Pretreatment. J. Agric. Food Chem. 61 (3), 635–645. 10.1021/jf3051939 23265413

[B48] Wen J.-L. Sun S.-L. Yuan T.-Q. Sun R.-C. (2015). Structural Elucidation of Whole Lignin from Eucalyptus Based on Preswelling and Enzymatic Hydrolysis. Green. Chem. 17 (3), 1589–1596. 10.1039/c4gc01889c

[B49] Wen J.-L. Sun S.-L. Yuan T.-Q. Xu F. Sun R.-C. (2014). Understanding the Chemical and Structural Transformations of Lignin Macromolecule during Torrefaction. Appl. Energ. 121, 1–9. 10.1016/j.apenergy.2014.02.001

[B50] Wen J.-L. Sun Y.-C. Xu F. Sun R.-C. (2010). Fractional Isolation and Chemical Structure of Hemicellulosic Polymers Obtained from Bambusa Rigida Species. J. Agric. Food Chem. 58 (21), 11372–11383. 10.1021/jf1032153 20942388

[B51] Wen J.-L. Xue B.-L. Xu F. Sun R.-C. Pinkert A. (2013). Unmasking the Structural Features and Property of Lignin from Bamboo. Ind. Crops Prod. 42, 332–343. 10.1016/j.indcrop.2012.05.041

[B52] Yang L. Lin M. Zhang H. Wang C. Shi L. Lan W. (2021). Ferulate-Sinapyl Alcohol Cross-Coupling Reaction Improves the Understanding of Grass Cell Wall Lignification. Ind. Crops Prod. 168, 113587. 10.1016/j.indcrop.2021.113587

[B53] Yuan T.-Q. Xu F. He J. Sun R.-C. (2010). Structural and Physico-Chemical Characterization of Hemicelluloses from Ultrasound-Assisted Extractions of Partially Delignified Fast-Growing Poplar Wood through Organic Solvent and Alkaline Solutions. Biotechnol. Adv. 28 (5), 583–593. 10.1016/j.biotechadv.2010.05.016 20493941

[B54] Zhao C. Hu Z. Shi L. Wang C. Yue F. Li S. (2020). Profiling of the Formation of Lignin-Derived Monomers and Dimers from Eucalyptus Alkali Lignin. Green. Chemi 22 (21), 7366–7375. 10.1039/d0gc01658f

[B55] Zhao X. Zhang L. Liu D. (2012). Biomass Recalcitrance. Part I: The Chemical Compositions and Physical Structures Affecting the Enzymatic Hydrolysis of Lignocellulose. Biofuels, Bioprod. Bioref. 6 (4), 465–482. 10.1002/bbb.1331

[B56] Zheng Y. Yu Y. Lin W. Jin Y. Yong Q. Huang C. (2021). Enhancing the Enzymatic Digestibility of Bamboo Residues by Biphasic Phenoxyethanol-Acid Pretreatment. Bioresour. Technol. 325, 124691. 10.1016/j.biortech.2021.124691 33461121

[B57] Zhou C.-H. Xia X. Lin C.-X. Tong D.-S. Beltramini J. (2011). Catalytic Conversion of Lignocellulosic Biomass to Fine Chemicals and Fuels. Chem. Soc. Rev. 40 (11), 5588–5617. 10.1039/c1cs15124j 21863197

[B58] Zhu J. Y. Pan X. J. (2010). Woody Biomass Pretreatment for Cellulosic Ethanol Production: Technology and Energy Consumption Evaluation☆. Bioresour. Technol. 101 (13), 4992–5002. 10.1016/j.biortech.2009.11.007 19969450

