# Supplementary material for: Effects of P-Coumarate 3-Hydroxylase Downregulation on the Compositional and Structural Characteristics of Lignin and Hemicelluloses in Poplar Wood (Populus alba × Populus glandulosa)
Source: Front Bioeng Biotechnol. 2021 Nov 15;9:790539. doi: 10.3389/fbioe.2021.790539 (PMC8634402; doi:10.3389/fbioe.2021.790539)
Supplement: Supplementary file 1 [file DataSheet1.docx]

**Effects of P-coumarate 3-hydroxylase (C3H) down-regulation on the compositional and structural characteristics of lignin and hemicelluloses in Populus alba × Populus glandulosa**

Xiao-Peng Peng^1^, Jing Bian^2^, Shuang-Quan Yao^3^, Cheng-Ye Ma^2*^, Jia-Long Wen^2*^

^1^ State Key Laboratory of Tree Genetics and Breeding, Key Laboratory of Tree Breeding and Cultivation of the National Forestry and Grassland Administration, Research Institute of Forestry, Chinese Academy of Forestry, Beijing 100091, China.

^2^ Beijing Key Laboratory of Lignocellulosic Chemistry, Beijing Forestry University, Beijing 100083, China

^3^ Guangxi Key Laboratory of Clean Pulp & Papermaking and Pollution Control, College of Light Industry and Food Engineering, Guangxi University, Nanning 530004.

Corresponding author at:

Beijing Key Laboratory of Lignocellulosic Chemistry, Beijing Forestry University, Beijing, 100083, China. E-mail addresses: [chengye.ma@foxmail.com](mailto:chengye.ma@foxmail.com) (C.-Y. Ma); wenjialong@bjfu.edu.cn (J.-L. Wen).

**1. The construction of RNAi inhibitory expression vector pBIRNAi-C3HR-i-C3HL**

In this study, we constructed the RNAi inhibitory expression vector pBIRNAi-C3HR-i-C3HL. We weigh the xylem material 0.2g, grinding it in liquid nitrogen into powder, and use RNA extraction kit. According to the instructions of the steps to operate, the separation of RNA dissolved in 60μL without RnaseddH2O, and get DNase removal of residual DNA.RNA purification process is as follows: Take 20 μL of the isolated RNA in a 0.5 mL centrifuge tube, and add double distilled water to 42 μL. Then add 5 μl of 10 × DNase reaction buffer, 1 μL of the enzyme inhibitor (40 U / mL), 2μL DNase (Rnase-free 1U / μL), 37 ℃ temperature bath 20 ~ 30min. Next, add 50 μL of ddH2O without Rnase, and add 100 μL of phenol / chloroform / isoamyl alcohol (ratio 25: 24:1).Mix it well and centrifuge it at 12000 rpm for 10 min. The supernatant was transferred to a new tube and10 μL of 3M NaOAC (pH = 5.2) was added. 250 μL of absolute ethanol was added, too. The mixture was allowed to stand at -20℃ for 40 min. After that, the precipitate was recovered and washed with 70% ethanol, centrifuged at 12000 rpm for 10 min by vacuum drying, and dissolved in 10 μlddH2Owithout Rnase, and confirmed by electrophoresis at 65 ° C for 5 min.

**2. Determination method of Phloroglucinol**

Put the sliced material onto the slide and add a drop of 25% HCL to impregnate material. Under acidification, the material was added a drop of phloroglucinol ethanol solution (1% of the pyrogallol 95% ethanol solution). Close the coverslip and observe. Bar6.13um lignified cell wall showed red reaction, and the color will deepen with the increased degree of lignification. Therefore, it was concluded that the transgenic C3H lignin content was lower than CK. Transgenic C3H lignin content is less than CK. The transgenic C3H’s catheter is more than CK. Fewer young secondary wall thickening.


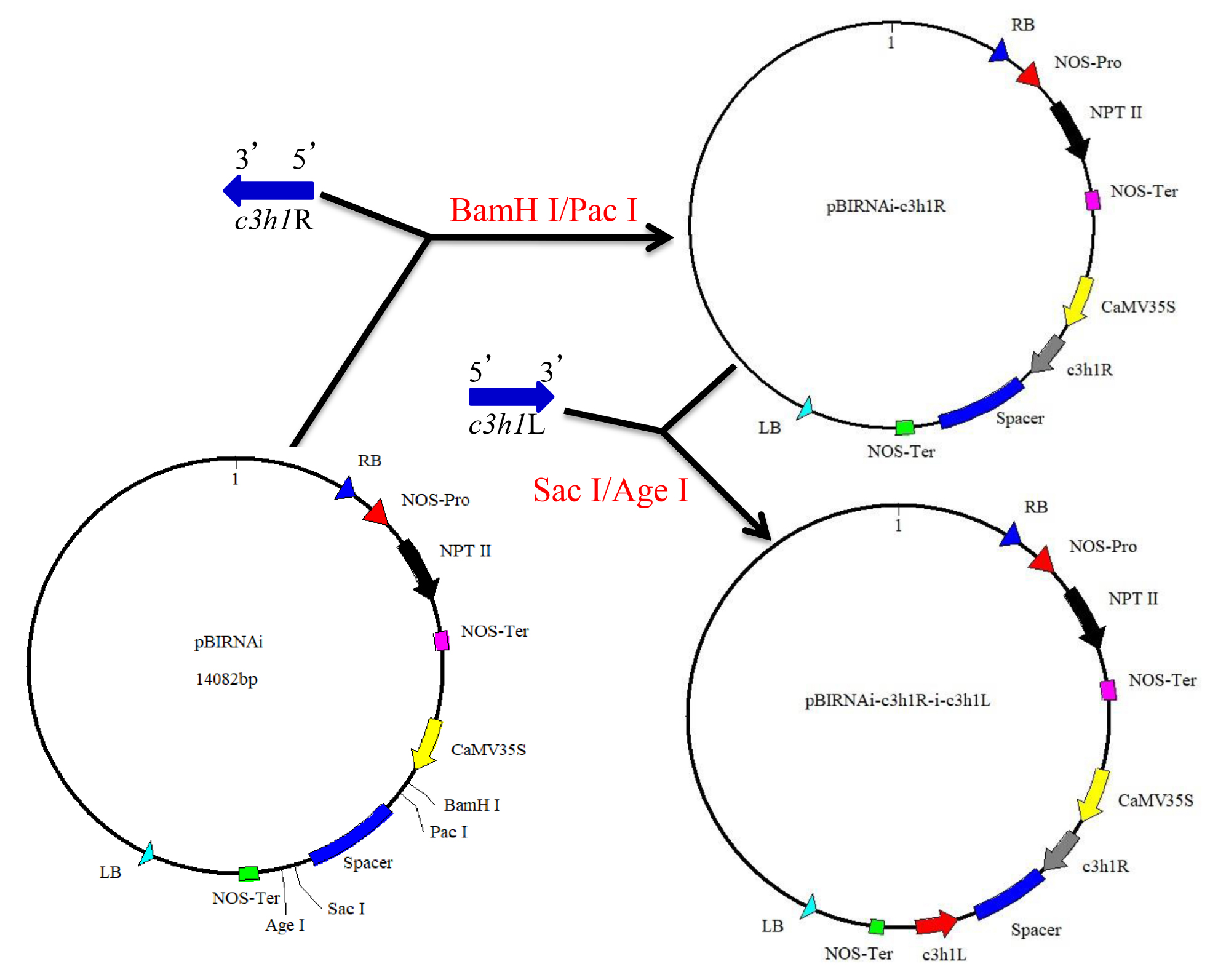
 Fig. S1 Construction flow chart of vector pBIRNAi-C3HR-i-C3HL


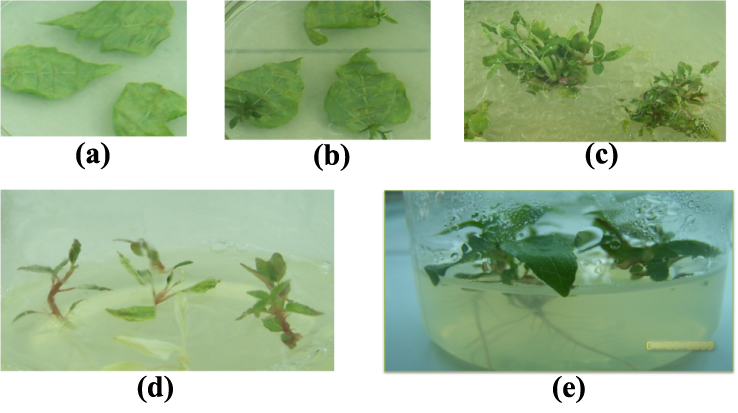
Fig. S2 Poplar genetic transformation via leaf dish method; (a) co-cultivation; (b) callus induction; (c) and (d) shoots resistant to kanamicine; (e) rooting of the shoots.


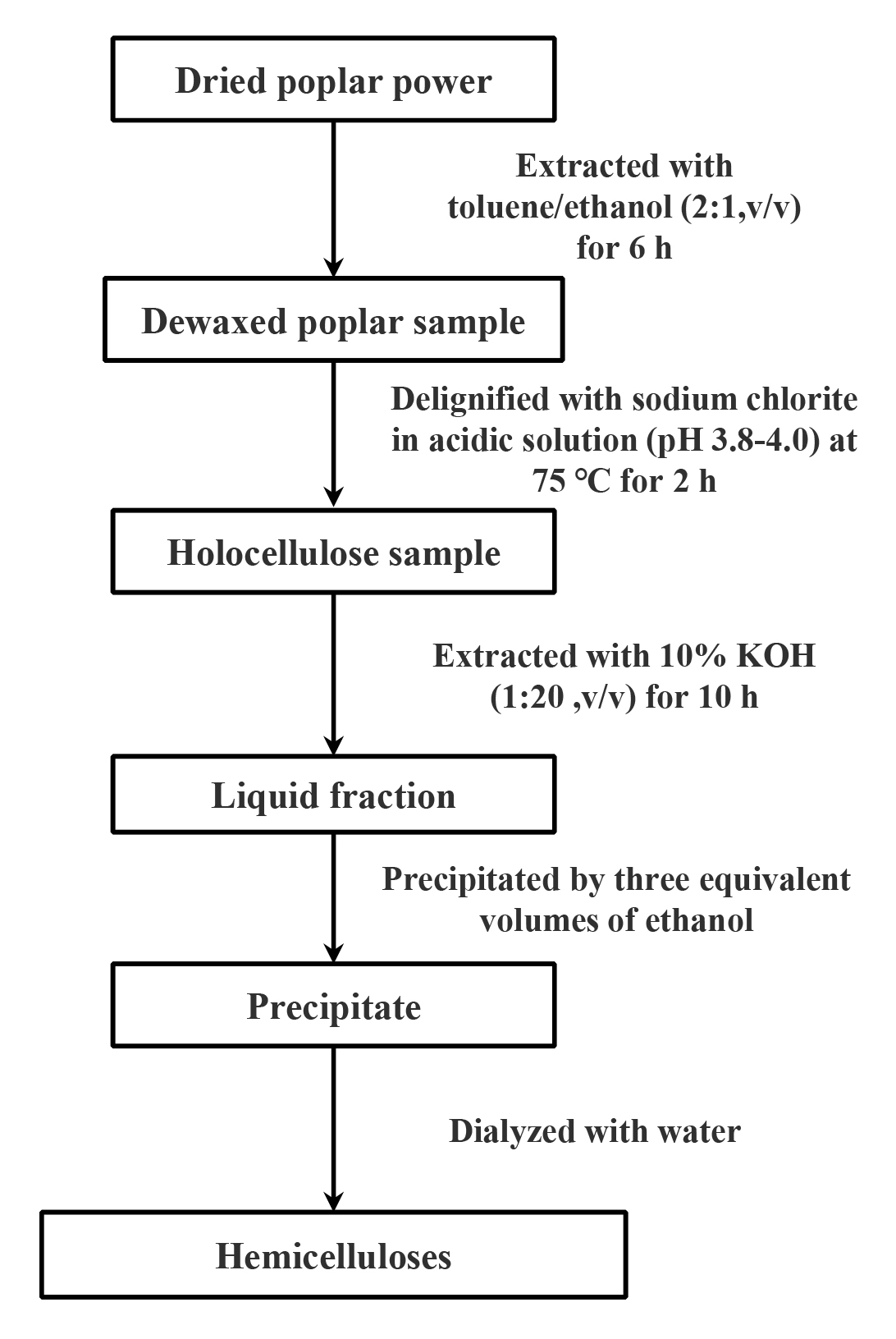
Fig. S3 The scheme of hemicelluloses isolation

Fig
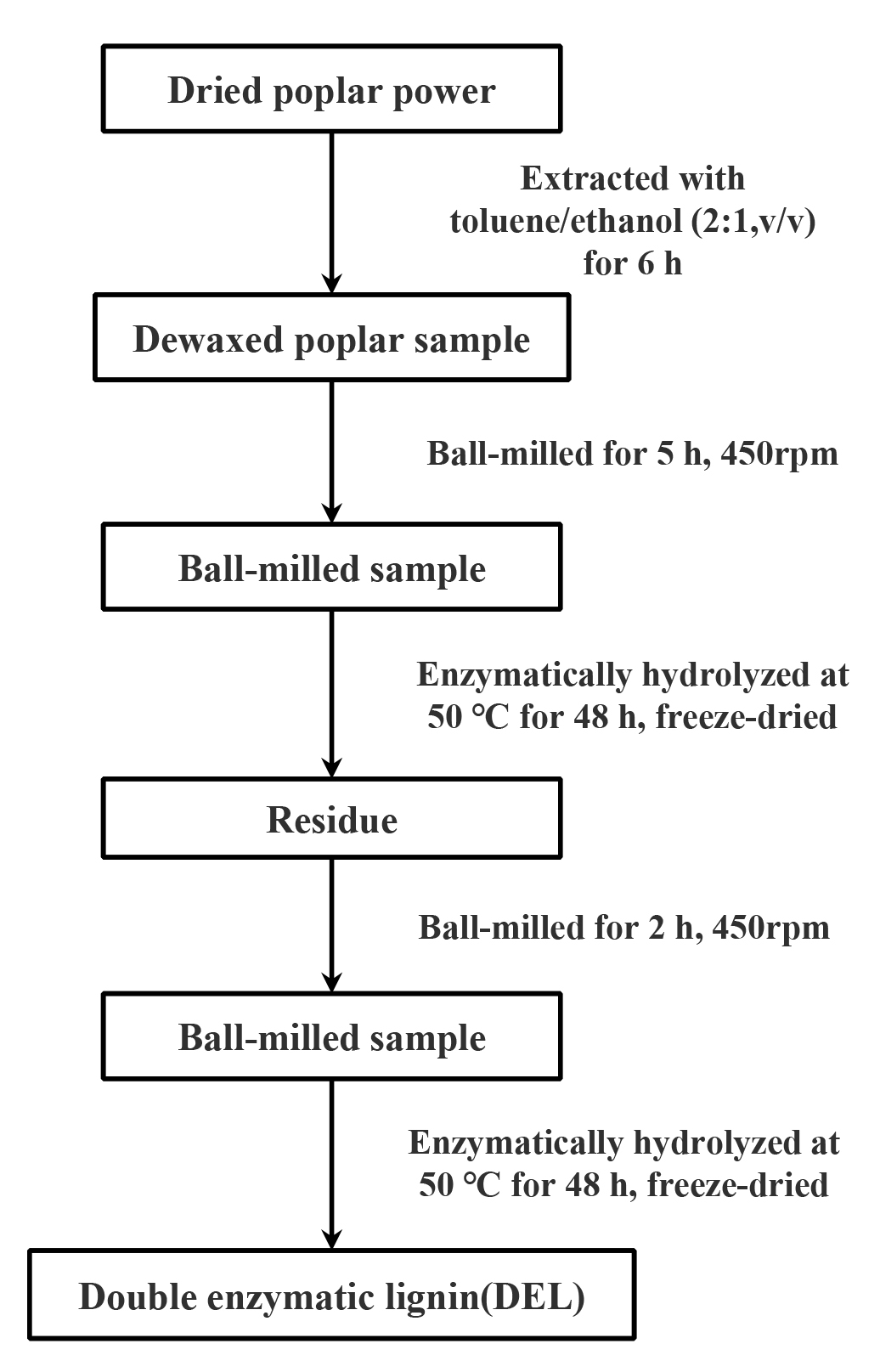
. S4 The scheme of native double enzymatic lignin (DEL) preparation





Fig. S5 Transcriptional abundances of C3H in transgenic plants (C3H) and wild type (CK).
